# Efficacy of Simplifying Complex Insulin Regimen on Glycometabolic Parameters and Target Organ Damage in Type 2 Diabetes: A Retrospective Cohort Study

**DOI:** 10.1155/jdr/9141564

**Published:** 2025-04-15

**Authors:** Roland Fejes, Csilla Kádár, Róbert Kovács-Huber, Zoltán Taybani, László Juhász, Attila Rutai, Szabolcs Péter Tallósy

**Affiliations:** ^1^Institute of Surgical Research, Albert Szent-Györgyi Medical School, University of Szeged, Szeged, Hungary; ^2^Department of Internal Medicine, Hódmezővásárhely-Makó Healthcare Center, Makó, Hungary; ^3^1st Department of Endocrinology, Dr. Réthy Pál Member Hospital, Békés County Central Hospital, Békéscsaba, Hungary

**Keywords:** liraglutide, lixisenatide, macrovascular complication, microvascular complication, retrospective study, therapy simplification, Type 2 diabetes

## Abstract

**Background:** Fixed-ratio combinations (FRCs) provide an alternative to intensified conservative insulin treatments (ICTs); however, therapy simplification in patients with high total daily insulin dose (TDD) or high HbA_1c_ is a debated issue; additionally, its influence on target organ damage (TOD) is less known.

**Methods:** Data were retrospectively collected from patients with Type 2 diabetes, including 58 patients who continued ICT and 104 patients who underwent therapy simplification between January 1, 2017, and January 1, 2023. Patient characteristics and therapy details are at baseline and 3, 6, 12, and 24 months after FRC initiation.

**Results:** HbA_1c_ significantly decreased in both groups (−0.9% [−1.6%, −0.5%] with ICT vs. −1.3% [−2.1%, −0.3%] with FRC), whereas body weight significantly decreased only after simplification (−1 kg [−4, 1] vs. −5 kg [−7, −2]). Diabetes duration was not associated with therapy efficacy. Significant HbA_1c_ reduction and FRC dose elevation occurred earlier in patients with an initial HbA_1c_ > 8.0% than in those with an initial HbA_1c_ < 8.0%. FRC dose was significantly higher at 3 months in patients with a TDD of > 60 U/day than in those with lower TDD. Relative risk reduction with therapy simplification was 72.1%, 50.6%, 32.3%, and 59.7% for hypoglycemia, renal function decline, microalbuminuria, and macrovascular complications, respectively. Risk of retinopathy, neuropathy, and chronic kidney disease did not significantly change with FRCs.

**Discussion:** FRCs are safe and as effective as ICT even in patients with high initial HbA_1c_, high TDD, or long diabetes duration. A protective role of FRCs in diabetic ASCVD has been proven, but their protective role in CKD was not observed.

**Conclusions:** The significant improvements in glycemic and weight control, as well as in TODs, suggest that therapy simplification may represent a more favorable approach compared to the continuation of previous ICT even in patients characterized by high baseline TDD and HbA_1c_ levels.

## 1. Introduction

For a considerable time, the traditional treatment hierarchy for type 2 diabetes (T_2_D) has included intensified conservative insulin therapy (ICT) for patients in whom relevant therapeutic modifications are no longer possible. ICT achieves rapid euglycemia [1]. However, the risk of weight gain and hypoglycemia as well as the rigid therapeutic regimen aggravates patient dissatisfaction, posing major disadvantages for ICT and may lead to ineffective therapy [1, 2]. Glucagon-like peptide 1 receptor agonists (GLP-1RAs) exert cardiovascular (CV) benefits [3–6]. The development of fixed-ratio combinations (FRCs) of GLP-1RAs and basal insulins has been a fundamental breakthrough in the therapeutic arsenal for T_2_D [7], although the introduction of FRCs has allowed the simplification of previously considered definitive ICTs [8].

Two FRC formulations are available on the market: IDegLira, including insulin degludec and liraglutide, and iGlarLixi, including insulin glargine and lixisenatide. GLP-1RAs and basal insulins exhibit a synergistic effect with higher safety in comparison to single-injection basal insulin and ICT regimens [9, 10]. Due to the progressive nature of diabetes, the efficacy of therapy simplification may be impacted not only by diabetes-related factors, such as insulin requirement, glycated hemoglobin (HbA_1c_) level, and body weight (BW), but also by the disease duration. The main gaps in knowledge regarding the efficacy of therapy simplification are especially overt in specific patient populations, such as those with high HbA_1c_ levels or high insulin requirements. Although beneficial CV profile of GLP-1RAs is well recognized, the impact of FRCs on diabetes-associated micro- and macrovascular target organ damages (TODs) is unclear, as no aimed clinical study can be found in English literature. The presence of TOD serves as an independent risk factor for the development of CV complications, irrespective of other existing risk factors. Therefore, the prevention and management of TODs are of paramount importance. Through its GLP-1RA component, therapy simplification may help slow the progression of, or even prevent, TOD [3–6]. Although the introduction of FRCs occurred roughly 10 years ago, national and international societies include therapy simplification with varying degrees of significance in their recommendations, and there is a paucity in prospective clinical trials aimed at addressing these gaps in knowledge [11–13].

In the present study, we aimed to retrospectively examine the efficacy and safety of therapeutic regimen simplification using iGlarLixi or IDegLira over a 24-month observation period using real-world data, including patients with high total daily insulin dose (TDD, > 60 U/day), high initial HbA_1c_ (> 8.0%), and long diabetes duration. A further major objective of this work was to investigate the advantages of FRCs over ICT therapy regarding diabetes-associated TODs, thereby providing entirely novel findings to substantiate and reinforce the benefits of this existing therapeutic approach.

## 2. Materials and Methods

### 2.1. Study Design, Subjects, and Ethical Considerations

This was a retrospective study based on data collected in an outpatient diabetes clinic at a secondary healthcare hospital in Hungary. The study included Caucasian male and female patients over 18 years of age diagnosed with T_2_D, and all data were collected during the period between January 1, 2017, and January 1, 2023.

**Figure 1:**
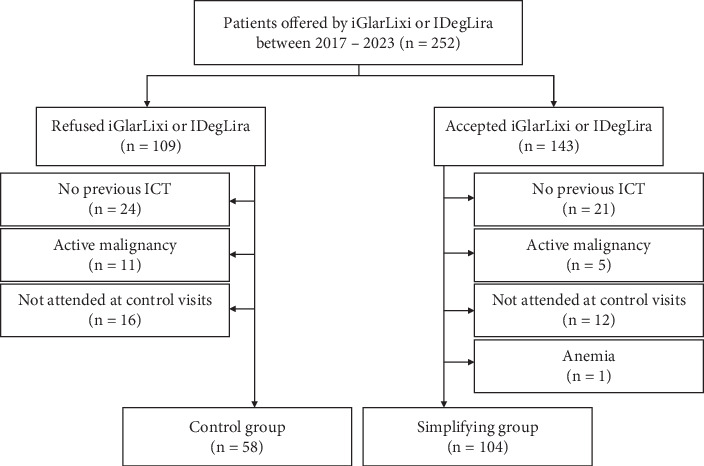
Schematic flowchart showing the inclusion and exclusion criteria used for patient selection. Inadequate follow-up meant a change of the treating physician or unexplained loss of contact with the patient within the first 6 months after the baseline visit.

The study cohort was divided into control (*n* = 58) or simplification (*n* = 104) groups. Therapy simplification was recommended due to the high risk of hypoglycemia, patient's desire for weight loss or more flexible therapy, and impaired quality of life due to frequent needle sticks. The control group included patients who continued previously initiated ICT ± oral antidiabetic drugs (OADs) after their refusal to switch to FRCs. Reasons of refusal comprised mostly financial causes or desired habits with the old therapy. The simplification group included patients who were initiated iGlarLixi or IDegLira for therapy simplification; both FRCs were included to cover the whole therapeutic palette. Therapy simplification was defined as a complete switch from ICT ± OADs to FRC ± OADs. Patients who used more than two OAD types were excluded. All patients in both groups had to be prescribed metformin as one of the OADs. Exclusion criteria included FRC prescription in an escalating therapeutic strategy, hemoglobin level of < 100 g/L, and active malignancy. Study flowchart is shown in Figure 1. The first outpatient visit, during which therapy simplification was initiated, was considered the baseline visit (BV), and data from the BV were considered as baseline values. The study was conducted in accordance with the 2008 revised Helsinki Declaration.

The study protocol was approved by the Institutional Review Board of Hódmezővásárhely-Makó Healthcare Center and the Hungarian National Public Health Center Institutional Committee of Science and Research Ethics (NNGYK/GYSZ/293-2/2024). All data were fully anonymized. The study was recorded and presented according to the requirements of the STROBE checklist.

### 2.2. Data Collection

Data were collected at the BV and at 3-, 6-, 12-, and 24-month visits. For all patients, the following data were collected: sex, age, height, BW, diabetes duration, types and doses of insulins used before therapy simplification, and FRC doses. TDD was expressed as U/day or U/kilogram/day. Body mass index (BMI) was calculated as the quotient of BW and the square of height (kilogram/square meter). Time dependence was determined by calculating parameter changes at specific timepoints compared to the values at the BV (*Δ*). Adverse events and TOD were also recorded. Diabetic retinopathy was diagnosed by an ophthalmologist. Peripheral sensory neuropathy diagnosis was based on impaired touch, pinprick, and vibration sensation with typical complaints and improvement with alpha-lipoic acid therapy. Atherosclerotic cardiovascular disease (ASCVD) includes coronary heart disease (myocardial infarction, angina, coronary artery stenosis), cerebrovascular disease (transient ischemic attack, ischemic stroke, carotid artery stenosis), and aortic and peripheral atherosclerotic disease (claudication, aortic aneurism). Elevated urine albumin/creatinine ratio (UACR) was based on a cutoff value of > 30 mg/g or 3 mg/mmol. Chronic kidney disease (CKD) was defined as an estimated glomerular filtration rate (eGFR) of > 60 mL/min/1.73 m^2^ for > 3 months. Hypoglycemia was defined as a blood glucose level of < 3.9 mmol/L. Hypoglycemia was recorded based on self-monitored glucose levels included in medical records.

### 2.3. Statistical Analysis

All analyses were conducted using the SigmaStat 13 software (Systat Software, San Jose, California, United States). Normality of the data was assessed using the Shapiro–Wilk test. Normally distributed continuous data were presented as means ± SD, and nonnormally distributed continuous data were presented as medians with 25th and 75th percentile values. Categorical data were presented as frequencies with numbers. Two-sample Student's *t*-test was applied to compare continuous numerical variables, while categorical variables and frequency differences were analyzed using the chi-square test. Differences between groups in the follow-up setups were evaluated using one-way analysis of variance (ANOVA) with the Holm–Sidak *post hoc* test or the Kruskal–Wallis one-way ANOVA by ranks followed by Dunn's method, depending on the data distribution. Correlation analyses were performed using Pearson's method. *p* values of < 0.05 were considered to indicate statistical significance. Risk for TOD and adverse events was expressed as proportions, relative risk (RR), relative risk reduction (RRR), absolute risk reduction (ARR), number needed to treat (NNT), and odds ratio (OR) with 95% confidence intervals (CIs). Parameters for TOD and adverse event outcomes were calculated using the standard methods, described in Supporting Information S1.

## 3. Results

### 3.1. Patient Characteristics at Baseline

From a total of 252 patients with available data, 109 refused and 143 accepted therapy simplification. A total of 90 patients were excluded according to the study criteria, and the remaining 58 and 104 patients were included in the control and simplification groups, respectively (Figure 1). The patient characteristics at BV were not significantly different either between the control and simplification (Table 1) or between iGlarLixi and IDegLira groups (Table S2).

### 3.2. Changes in HbA_1c_ Levels, BW, and FRC Doses Over Time

**Figure 2:**
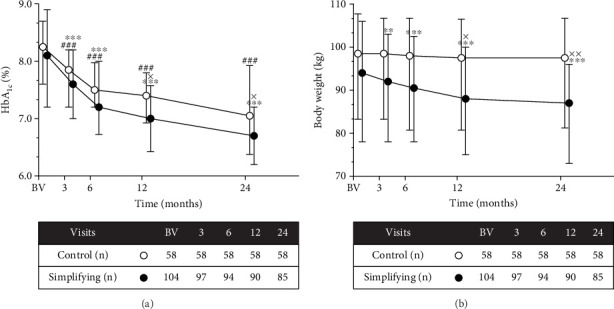
Changes in HbA_1c_ (a) and body weight (b). Data are displayed as medians with 25th and 75th percentiles, indicated as straight lines. Open circles indicate the control group, and black full circles indicate the therapy simplification group. Comparison of data with baseline visit (BV) was performed using repeated measures analysis of variance followed by Dunn's method. Comparison between groups within visits was performed using Student's *t -*test. ^#^*p* < 0.05, ^##^*p* < 0.01, ^###^*p* < 0.001 vs. BV in the control group. ⁣^∗^*p* < 0.05, ⁣^∗∗^*p* < 0.01, ⁣^∗∗∗^*p* < 0.001 vs. BV in the simplifying group. ^x^*p* < 0.05, ^xx^*p* < 0.01, ^xxx^*p* < 0.001 vs. control within each timepoint.

HbA_1c_ and BW significantly declined in the simplification group (*p* < 0.001 for both at 24 months), whereas a significant decline at 24 months was observed only in HbA_1c_ in the control group (*p* < 0.001, Figure 2). At the 24-month visit, the HbA_1c_*Δ* was −0.85% (−1.62%, −0.47%) in the control group and −1.3% (−2.07%, −0.30%) in the simplification group, and HbA_1c_ levels were significantly lower in the simplification group than in the control group at 12- and 24-month visits (*p* = 0.018 and *p* = 0.039, respectively). BW was significantly lower in the simplification group than in the control group at both 12- and 24-month visits (*p* = 0.025 and *p* = 0.006, respectively). The median BW*Δ* at 24 months was −5.0 kg (−7.0, −2.0) in the simplification group and −1.0 kg (−4.0, 1.0) in the control group. In the control group, at the 24-month visit, HbA_1c_ reductions of > 1.0% and 0.0%–1.0% were observed in 23 patients (39.6%) and 24 (41.3%) patients, respectively, whereas a slight HbA_1c_ elevation was observed in 4 (6.89%) patients. Conversely, in the simplification group, at the 24-month visit, HbA_1c_ reductions of > 1.0% and 0.0%–1.0% were observed in 45 (52.9%) and 25 (32.9%) patients, respectively, whereas HbA_1c_ did not decline in 12 (14.1%) patients. At the 24-month visit, 29 patients (50.0%) from the control group and 57 patients (54.8%) from the simplification group reached the 7.0% HbA_1c_ level. Sustained weight loss was achieved in 78 patients (75.0%) at the 24-month visit. The ICT and FRC doses significantly increased during the study period (*p* < 0.001 for both), culminating at an ICT dose of 60.0 U/day (77.7, 50.5) in the control group and an FRC dose of 22.0 U/day (18.0, 27.0) in the simplification group at the 24-month visit. Supporting Information S3 provides a brief comparison of the effects of iGlarLixi and IDegLira on major variables.

### 3.3. Changes in HbA_1c_ Levels, BW, and FRC Doses Over Time According to the Initial HbA_1c_ Level

**Figure 3:**
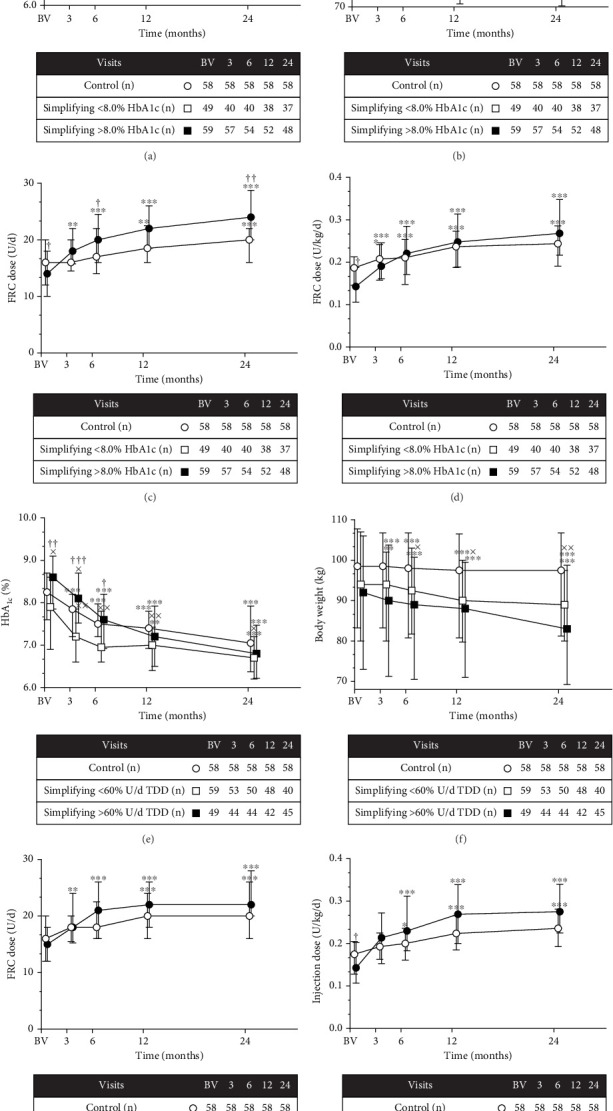
Changes in HbA_1c_ (a), body weight (b), and fixed-ratio combination (FRC) doses (c, d) according to initial HbA_1c_. Changes in HbA_1c_ (e), body weight (f), and FRC doses (g, h) according to total daily insulin dose (TDD). All data are displayed as medians with 25th and 75th percentiles, indicated as straight lines. Patients with low and high initial HbA_1c_ or TDD were also compared. Comparisons between subgroups within specific timepoints were conducted using the Mann–Whitney *U* test, and analyses within subgroups compared to the baseline visit (BV) were performed using repeated measures analysis of variance test followed by Dunn's method. ⁣^∗^*p* < 0.05, ⁣^∗∗^*p* < 0.01, ⁣^∗∗∗^*p* < 0.001 vs. BV within groups. ^†^*p* < 0.05, ^††^*p* < 0.01, ^†††^*p* < 0.001 vs. control within visits. ^x^*p* < 0.05, ^xx^*p* < 0.01, ^xxx^*p* < 0.001 vs. > 8.0% HbA_1c_ or > 60 U/day TDD group within visits.

The patients in the simplification group were further categorized into those with high and low initial HbA_1c_ levels based on a BV HbA_1c_ cutoff of 8.0% (Figures 3a, 3b, 3c, and 3d). HbA_1c_ exhibited a significant decline at the 6-, 12-, and 24-month visits compared to the BV in the high initial HbA_1c_ group (*p* < 0.001 for all), whereas the decline in HbA_1c_ from the BV became significant only at the 24-month visit in the low initial HbA_1c_ group (*p* = 0.005, Figure 3a). At the 24-month visit, the high initial HbA_1c_ group reached the level of the control and low initial HbA_1c_ groups (Figure 3a). From the total of 57 patients, who reached the 7.0% HbA_1c_ level at the 24-month visit, 26 patients (45.6%) were from the low initial HbA_1c_ group and 31 patients (54.4%) from the high initial HbA_1c_ group. BW decreased significantly at all timepoints compared to the BV values in both HbA_1c_ subgroups (low initial HbA_1c_: *p*_3mo_ = 0.01, *p*_6,12,24mos_ < 0.001; high initial HbA_1c_: *p*_6,12,24mos_ < 0.001). The BW*Δ* during the study period was not significantly different between the control and the high initial HbA1c group, although BW was significantly lower in the low HbA_1c_ group than in the control group at the 12 and 24-month visits (*p* = 0.007 and *p* = 0.002, respectively, Figure 3b). FRC doses (U/day) were significantly higher at all visits than at the BV in the high initial HbA_1c_ subgroup (*p*_3mo_ = 0.004, *p*_6,12,24mos_ < 0.001) and at the 12- and 24-month visits than at the BV in the low initial HbA_1c_ group (*p* = 0.003 and *p* < 0.001, respectively). Significant differences were observed in FRC doses at the BV and at the 6- and 24-month visits between the subgroups (*p* < 0.001, Figure 3c). Interestingly, FRC doses (U/kilogram/day) were not significantly different between the subgroups at the case of comparison with the U/kilogram/day dimension (Figure 3d).

### 3.4. Changes in HbA_1c_ Levels, BW, and FRC Doses Over Time According to TDD

We also performed analyses in patients categorized into high and low TDD groups based on an initial TDD cutoff of 60 U/day in the simplification group. HbA_1c_ levels were significantly lower at the 12- and 24-month visits than at the BV in the low TDD subgroup (*p* = 0.007 and *p* < 0.001, respectively). Similarly, HbA_1c_ was significantly lower at the 6-, 12-, and 24-month visits than at the BV in the high TDD subgroup (*p* < 0.001 at all, Figure 3e). HbA_1c_ was significantly lower in the low TDD group than in the control group (*p*_3mo_ = 0.003, *p*_6mo_ = 0.009, *p*_12mo_ = 0.006, *p*_24mo_ = 0.048; Figure 3e). From the total of 57 patients, who reached the 7.0% HbA_1c_ level at the 24-month visit, 36 patients (63.2%) were from the low TDD group and 21 patients (36.8%) from the high TDD group. BW decreased significantly in both subgroups (low TDD: *p*_6,12,24mos_ < 0.001; high TDD: *p*_6,12,24mos_ < 0.001). Compared to the control group, BW was significantly decreased in the low TDD group at 6, 12, and 24 months (*p* = 0.048, *p* = 0.02, and *p* = 0.006, respectively) but not in the high TDD group (Figure 3f). FRC doses (U/day) were significantly increased only at the 12- and 24-month visits in the low TDD subgroup compared to the BV (*p* < 0.001 at both), whereas significant increases in FRC doses occurred at earlier timepoints in the high TDD subgroup (*p*_3mo_ = 0.005, *p*_6,12,24mos_ < 0.001; Figure 3g,h).

### 3.5. Associations Among the Major Study Parameters

**Figure 4:**
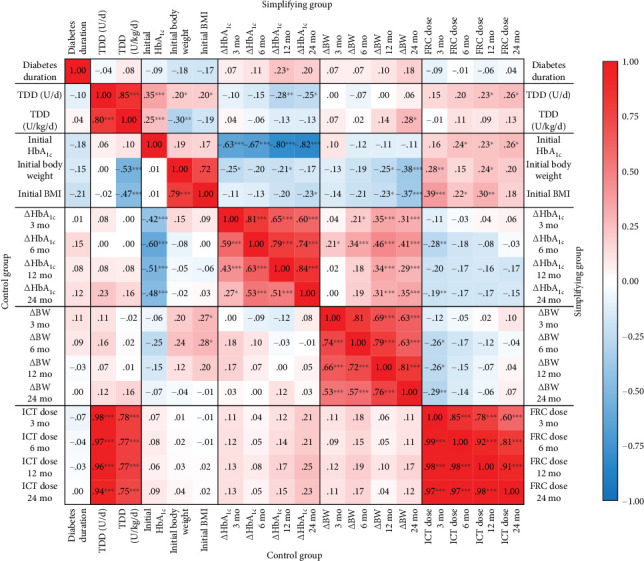
Correlation matrix showing the relationships between initial and follow-up parameters during the study period. The color scheme of the heat map is based on correlation coefficients, which is indicated in every block of the matrix. Correlation analysis was performed using Pearson's method. ⁣^∗^*p* < 0.05; ⁣^∗∗^*p* < 0.01; ⁣^∗∗∗^*p* < 0.001.

Evaluation of the associations among the major study parameters displayed as a correlation matrix with correlation coefficients can be seen in Figure 4. We did not observe an association between diabetes duration and other parameters in either the control or the simplification group. In the control group fair, inverse correlations were observed between HbA_1c_*Δ* and initial HbA_1c_ at all visits (*r* ranges between −0.42 and −0.60, *p* < 0.001 for all). However, BW*Δ* was correlated with initial BMI at the 3- and 6-month visits (*r* = 0.27 with *p* = 0.039 and *r* = 0.28 with *p* = 0.033, respectively); their isolated nature and the low strength of correlations suggested a weak clinical relevance. Conversely, ICT doses, which were strongly and positively correlated with the initial TDD (*r* ranges between 0.75 and 0.98, *p* < 0.001 for all), did not exhibit correlations with other relevant parameters in the control group. In the simplification group, TDD exhibited weak, isolated correlations with the evaluated parameters. Similar to that observed in the control group, an inverse correlation was observed between HbA_1c_*Δ* and initial HbA_1c_ at all visits, although the observed associations were stronger. Although the correlations of BW and BMI with BW*Δ* were stronger in the simplification group than in the control group, the correlation coefficient remained weak even at the 24-month visit. Changes in HbA_1c_ exhibited a weak correlation with changes in BW, whereas BW*Δ* and HbA_1c_*Δ* did not exhibit significant correlations with FRC doses.

### 3.6. Adherence, Hypoglycemia, and Progression of TOD

**Figure 5:**
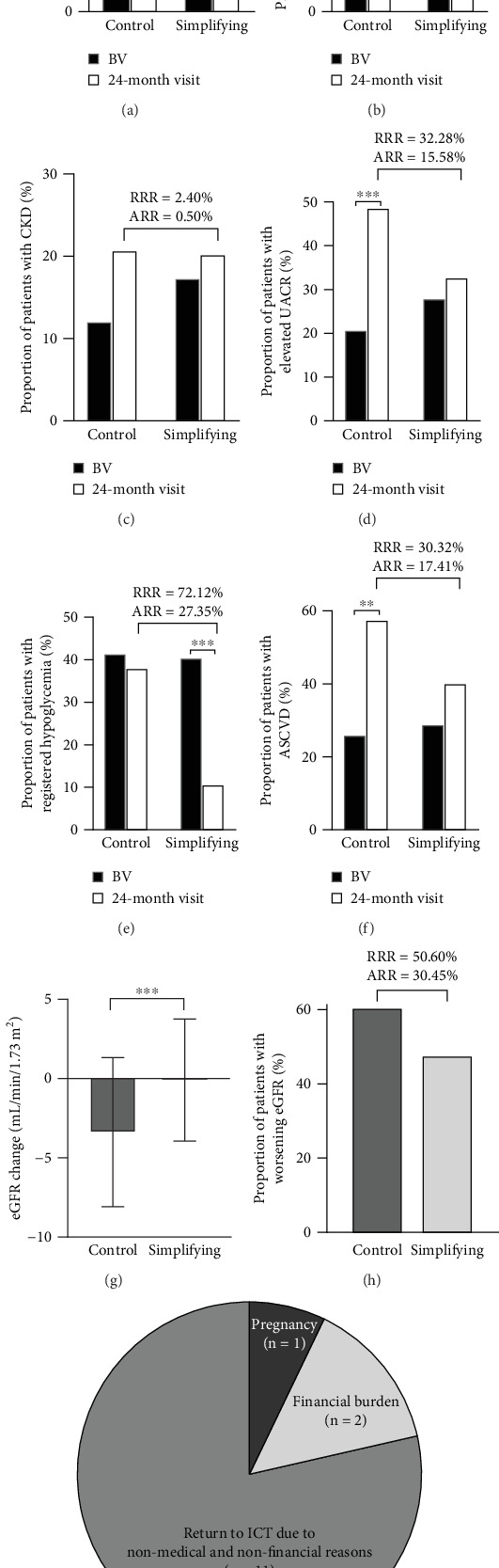
Risk of target organ damage and causes of therapy disruptions. (a) Retinopathy, (b) peripheral sensory neuropathy (PSN), (c) chronic kidney disease (CKD), (d) elevated urine albumin/creatinine ratio (UACR), (e) hypoglycemia, (f) therapy discontinuation, (g) change in estimated glomerular filtration rate (eGFR), (h) macrovascular complications, and (i) progressive eGFR decline.

Table S4 provides the rates, ARRs, RRs, RRRs, NNTs, and ORs, with 95% CIs for these parameters. The risk of retinopathy and PSN and the incidence of CKD were not significantly different between the two groups (Figures 5a, 5b, and 5c). Significant RRs were observed for elevated UACR (*p* = 0.0463, Figure 5d), hypoglycemia (*p* = 0.0001, Figure 5e), and ASCVD (*p* = 0.0383, Figure 5f). There was a significantly lower eGFR decrease in the simplification group (*p* < 0.001, Figure 5g) with a significantly lower risk for eGFR decrease (*p* < 0.001, Figure 5h). FRCs were discontinued in 14 (15%) patients; 11 patients requested a return to ICT due to dissatisfaction, two patients could not bear the additional financial burden, and one patient had to discontinue the FRC due to pregnancy (Figure 5i).

## 4. Discussion

Therapy simplification is a prevalent option in medicine; however, the application of FRCs within a simplifying regimen is an emerging approach. This research employed real-world data to assess the effectiveness of simplification using iGlarLixi and IDegLira, emphasizing changes in diabetes-associated TODs. We aimed to describe the influence of this approach on TOD progression, a topic that remains understudied. A further strength of this study is the inclusion of a control group, consisting of patients receiving ICT for comparison. Our primary goal was not to compare the two FRCs but to examine therapy simplification as an integrated therapeutic approach, as the available literature indicates no notable variations in the glycemic effectiveness and safety of the two FRCs [14, 15].

The normalization of BW and the reduction of HbA_1c_ are pivotal in managing diabetes. Extensive research has consistently highlighted the strong interrelation between weight loss and enhanced glycemic control, both of which have a substantial effect on the progression of TOD and overall life expectancy [7–10]. Our analyses revealed therapy simplification was linked to substantial decreases in HbA_1c_ levels (from 8.1% to 6.7%) and BW (from 94 to 87 kg). These results are aligned with existing literature; a brief comparison is provided in Supporting Information S7. More substantial and faster HbA_1c_ reductions were safely achieved in patients with elevated baseline HbA_1c_ levels, consistent with findings from the DUAL-HIGH Trial (NCT03737240) [16]. In the current research, FRCs were not inferior to ICT regarding HbA_1c_*Δ* as both therapeutic approaches resulted in an approximate from 1.0% to 1.5% HbA_1c_ reduction. There was only a slow and augmented decrease among the patient with a low BV HbA_1c_. Nevertheless, the observed modest effect cannot be considered neutral or disadvantageous. The majority of patients in this group was already within the target HbA_1c_ range; the primary therapeutic goal was not to further lower HbA_1c_, which would increase the risk of hypoglycemia [17–21], but to enhance safety and achieve weight loss. These effects were evident not only in patients with low BV HbA_1c_ but also in those with high BV HbA_1c_ levels. Regarding BW, simplification was superior with its 9-kg weight reduction to ICT, where no significant weight reduction was observed over the 24-month-long follow-up (from 98.5 to 97.5 kg). Weight loss is promoted by lower insulin doses in FRCs, facilitated by the insulin-sparing effect of GLP-1RAs, which also potentiate weight loss by their anorexigenic effect, as slowed gastric emptying or reduced appetite, which ultimately helps patients achieve and maintain a healthier BW [22]. This weight reduction is not only important for improving glycemic control but some studies reported a crucial role in long-term patient outcomes, particularly in lowering the risk of CV events, while enhancing overall metabolic health. Emphasizing these mechanisms can provide a more comprehensive insight into the wider benefits of GLP-1RAs beyond just blood glucose management [23, 24].

Notably, diabetes duration did not impact the efficacy expected with FRC therapy and the therapy simplification demonstrated success in patients regardless of TDD levels, whether low or high. Patients with longer disease duration usually need higher TDDs and are notably overweight [25–27]. It is possible that therapy simplification with FRCs is inherently doomed to failure in these individuals owing to high TDDs, because the insulin dose provided by FRCs may decrease to as much as one-third of the previous dose used with the ICT regimen. In a study by Szépkuti et al., IDegLira was more efficient than ICT in HbA_1c_ reduction [28]. Higher TDD at baseline is an indication of more advanced disease, against which GLP-1RAs perceived as less effective, since the enhancement of the endogenous insulin response is a major effect of GLP-1RAs. However, the rationale behind therapy simplification lies in the overuse of insulin therapy and the overtreatment of patients [7, 8]. Our data are not in line with this explanation, as all enrolled patients were insulin users. GLP-1RAs have glucose-lowering effects independent of insulinotropic properties [29–32], which cannot be ruled out.

The results on TODs were *per se* affected by the substantial pharmacological distinctions between liraglutide and lixisenatide. As our study design does not provide directly comparable data between these two agents, a detailed presentation of the ELIXA and LEADER (NCT01147250 and NCT01179048, respectively) studies, major CV outcome trials on these GLP-1RAs [23, 33], is available in Supporting Information S6. However, multiple studies have emphasized that comparing FRCs with stand-alone GLP-1RAs overlooks dosing differences of the GLP-1RA component [34–36]. Our results might be outstanding, as a thorough review of the English literature has not revealed clinical studies with a substantial sample size evaluating the impact of FRCs on TOD in comparison with ICT. A study by Cowart et al. is the only investigation comparing TOD between the two FRCs, where iGlarLixi was found superior to IDegLira in composite CV endpoints, heart failure, nephropathy, and neuropathy. However, no preferred selection recommendation currently exists from any major professional society [34].

Although preclinical studies show promising beneficial effects of GLP-1RAs in neuropathy and retinopathy, clinical studies report modest benefits in the progression of these microvascular complications [37–41]. Our findings are in line with the literature regarding PSN or retinopathy. Regarding renal endpoints, a significant RRR was detected in cases of elevated UACR whereas eGFR decline was significantly decelerated by therapy simplification. Furthermore, therapy simplification was linked to a substantial decrease in ASCVD risk (RRR 30.3%, 95% CI 1.93–50.49%, *p* = 0.0383), which may be ascribed to both the direct CV protective effects (e.g., blood pressure reduction or lipid profile normalization) of GLP-1RAs and their bidirectional interplay with other assessed target organ dysfunctions and glycometabolic parameters. Additionally, this effect may have been further impacted by lower daily insulin requirements and better BW management. The 72.1% RRR in hypoglycemic events while maintaining, or in certain instances even improving, glycemic control underlies the remarkable advantages of the simplifying regime. Several authors have acknowledged simplification for its key advantage of not only reducing the incidence of severe symptomatic hypoglycemia but also alleviating mild asymptomatic episodes, as documented in numerous studies through subcutaneous continuous glucose monitoring [23, 24, 42–45]. Beyond these medical benefits, multiple studies have demonstrated that using iGlarLixi or IDegLira enhances patients' quality of life (e.g., burden on social activities, pain due to treatment, worry about hypoglycemia or late complications) which leads to higher treatment adherence [42, 46]. Furthermore, several cost-effectiveness studies proved that despite their relatively high cost, iGlarLixi and IDegLira provide cost-effective treatment options over both the short and long term [11, 14, 47, 48]. However, it is worth mentioning that among the 14 patients who discontinued treatment, 11 requested a return to their prior regimen due to nonmedical or nonfinancial reasons. This psychic burden for ICT, in most cases, originated from a perceived lack of security stemming from their longstanding reliance on a four-times-daily injection schedule, often in place for a decade or more. Although we hypothesize that more comprehensive patient education could have further reduced this proportion, the findings suggest the safety and high tolerability of therapy simplification. Independent of the advantages of the FRCs, certain patient groups require special consideration, as the use of these agents may necessitate caution or be contraindicated. FRCs are not advised for patients lacking insulin secretion (proved by serum C-peptide level), advanced renal impairment (eGFR < 30 mL/min), pregnancy or breastfeeding, a history of pancreatitis, or known hypersensitivity to any components of these therapies [49]. Several studies have reported a varying degree of gastrointestinal side effects with FRCs. In the DUAL VII Trial (NCT02420262), IDegLira was compared with basal-bolus insulin therapy. Among the patients with IDegLira, 11.1% reported nausea, in contrast to only 1.6% of those on basal-bolus insulin therapy [50]. For iGlarLixi, nausea was observed in 9.6% of cases, compared to 3.6% with insulin glargine alone and 24.0% with lixisenatide alone [51]. Although the incidence of gastrointestinal side effects is significantly lower compared to stand-alone GLP-1RA therapies due to the much smaller lixisenatide and liraglutide doses—leading several expert reports to deem them safe in this regard—clinicians should exercise caution in individuals with a prior history of severe gastrointestinal irritability [52].

Our study has certain limitations warranting investigation. This work shares common shortcomings with single-center retrospective medical trials, including only a Caucasian population. Due to the comparatively low occurrence of TODs, the head-to-head analysis of FRCs had not yielded sufficiently reliable results. The influential role of that general medical practice cannot be discounted, where patients undergoing therapy changes receive increased attention from healthcare staff. An attempt to mitigate this factor was made by designating a 24-month follow-up period where this heightened attention no longer applied. However, the lack of results for certain TODs highlights the need for further research with an even longer follow-up. Furthermore, GLP-1RA was used as an add-on therapy, so the effects of nephron- and cardioprotective medications already in use before the study cannot be excluded (concomitant antidiabetic or nephron- and cardioprotective drug usage can be found in Table S5). Drug costs influence the clinical efficacy of a therapy. In this regard, the available literature is contradictory, but we did not have the opportunity to perform a cost-effectiveness analysis for additional support. It is worth noting the reporting of hypoglycemia, as self-monitoring of blood glucose by patients introduces a high potential for bias, such as hidden hypoglycemic events (e.g., oversleeping at night or in the morning). Continuous subcutaneous glucose monitoring systems present an alternative solution [53]. Looking ahead, building on the results of this work, our primary goal will be to organize a prospective study with a longer observation period; ideally, this study would involve multiple centers and a larger sample size, enabling us to comprehensively assess the development of a wide range of diabetes-related and other cardiorenal TODs.

In summary, the present study is aimed at broadening our understanding of T_2_D management, emphasizing the potential benefits and safety of therapy simplification with FRCs. The use of FRCs is not only safe but also as effective as ICT even in patients with high initial HbA_1c_ levels and those with TDDs. Our work is the very first comprehensive presentation of the effects of simplifying complex insulin regimen with FRCs on TODs. The protective role of FRCs in diabetic ASCVD proved to be outstanding, but to determine their potential protective role in CKD, further prospective studies are needed.

## Figures and Tables

**Table 1:** Clinicopathologic characteristics of patients enrolled in the study. Student's *t -*test was used for comparison of the control and simplification groups, and the chi-square test was used to compare the distribution of antidiabetic drug use between the groups. Data are presented as medians with 25th and 75th percentiles. *p* values of < 0.05 were considered to indicate statistical significance.

	**Control (** **n** = 58**)**	**Simplifying (** **n** = 104**)**	**p** ** value**
Age (years)	63.00 (57.00, 68.25)	63.00 (55.00, 69.00)	0.866
Diabetes duration (years)	11.00 (8.00, 14.00)	11.50 (4.00, 22.00)	0.951
Male (*n*, %)	31 (53.44%)	50 (48.07%)	0.036
HbA_1c_ (%)	8.25 (7.57, 8.75)	8.10 (7.20, 8.90)	0.991
HbA_1c_ (mmol/mol)	66.66 (59.28, 71.85)	65.02 (55.18, 73.76)	0.991
C-peptide (ng/mL)	4.05 (3.17, 4.92)	3.78 (2.59, 4.76)	0.205
Body weight (kg)	98.5 (82.75, 108)	94.00 (78.00, 106.00)	0.274
Body mass index (kg/m^2^)	30.27 (27.95, 33.88)	31.99 (27.27, 36.45)	0.389
Initial TDD (U/day)	55.00 (48.00, 77.00)	48.00 (40.00, 76.00)	0.0841
Initial TDD (U/day/kg)	0.62 (0.47, 0.75)	0.57 (0.43, 0.80)	0.398
Initial ICT with analog insulins (*n*, %)	35 (60.34%)	53 (50.96%)	0.250
Oral antidiabetics prior to simplifying (*n*, %)			
Metformin	50 (86.20%)	92 (88.46%)	0.6757
Sulfonylurea	15 (25.86%)	20 (19.23%)	0.325
DPP4i	23 (39.65%)	36 (34.61%)	0.522
GLP-1RA	5 (8.62%)	11 (10.57%)	0.689
SGLT2i	23 (39.65%)	31 (29.80%)	0.202
Concomitant drug use (*n*, %)			
RAASi	42 (72.41%)	67 (64.42%)	0.298
Beta blockers	26 (44.82%)	58 (55.77%)	0.181
MRA	14 (24.13%)	34 (32.69%)	0.252
Statins	33 (56.89%)	50 (48.07%)	0.140
Antiplatelet agents	20 (34.48%)	39 (37.50%)	0.702
Diabetes-related target organ damage prior to simplifying (*n*, %)			
eGFR (mL/min/1.73m^2^)	86.00 (70.00, 90.00)	87.50 (78.25, 90.00)	0.0577
CKD (*n*, %)	7 (12.07%)	18 (17.31%)	0.376
UACR > 30 mg/g (*n*, %)	12 (20.68%)	29 (27.88%)	0.312
Retinopathy (*n*, %)	13 (22.41%)	27 (25.96%)	0.615
PSN (*n*, %)	15 (25.86%)	29 (27.88%)	0.781
ASCVD (*n*, %)	17 (29.31%)	27 (25.96%)	0.646

## Data Availability

The data that support the findings of this study entitled “Efficacy of Simplifying Complex Insulin Regimen on Glycometabolic Parameters and Target Organ Damage in Type 2 Diabetes: A Retrospective Cohort Study” are available from the authors upon reasonable request and with permission from the corresponding ethical boards. Nonetheless, restrictions apply to the availability of these data, which were used after approval for the current study was obtained from the Institutional Review Board of Hódmezővásárhely-Makó Healthcare Center and the Hungarian National Public Health Center Institutional Committee of Science and Research Ethics.

## Supporting Information

Supporting Information Additional supporting information can be found online in the Supporting Information section. Supporting Information S1. In-depth description of the calculation of various statistical indicators on target organ damage and adverse effects outcomes. Proportions were used to express the frequency of outcomes, while relative risk (RR) and relative risk reduction (RRR) demonstrated the proportional differences between groups. Absolute risk reduction (ARR) provided the direct difference in event occurrence between the treatment and control groups. The number needed to treat (NNT) quantified how many individuals need to be treated to achieve one favorable outcome. The odds ratio (OR) was used to compare the relative odds of events, particularly for rare outcomes. All estimates were presented with 95% confidence intervals (CIs) to ensure the accuracy and reliability of the evaluations. Table S2. Clinicopathologic characteristics of patients in the simplification group at the BV visit. Patients were grouped based on whether therapy simplification was conducted with iGlarLixi or with IDegLira. Student's *t*-test and the chi-square test were used as appropriate. Data are presented as medians with 25th and 75th percentiles. *p* values of < 0.05 were considered to indicate statistical significance. Figure S3. Changes of major clinical parameters of patients in the simplification group from the BV to the 24-month visit. The figure shows HbA_1c_ (a), body weight (b), and fixed-ratio combination (FRC) doses in U/day (c) or in U/kilogram/day (d). Patients were grouped based on whether therapy simplification was conducted with iGlarLixi (empty circle) or with IDegLira (black circle). All data are displayed as medians with 25th and 75th percentiles, indicated as straight lines. Analyses within treatment arms compared to the baseline visit (BV) were performed using repeated measures analysis of variance test followed by Dunn's method. Comparisons between subgroups within specific time points were conducted using the Mann–Whitney *U* test and followed by Dunn's *post hoc* test. ⁣^∗^*p* < 0.05, ⁣^∗∗^*p* < 0.01, ⁣^∗∗∗^*p* < 0.001 vs. BV within groups. ⁣^x^*p* < 0.05, ⁣^xx^*p* < 0.01, ⁣^xxx^*p* < 0.001 vs. IDegLira within visit. Table S4. Risks of target organ damage and hypoglycemia between the control and the simplification group at the 24-month visit. Table S5. Changes in medication use at BV and at the 24-month visit. Comparison was conducted between control and simplification group within visit and between BV and 24-month visit within the treatment group by the chi-square test. Supporting Information S6. Comparison of cardiorenal effects of liraglutide and lixisenatide with a brief comparison of LEADER and ELIXA cardiovascular outcome trials. Supporting Information S7. Representative comparison of publications examining the effects of IDegLira or iGlarLixi in a simplifying approach.
