# Supplementary material for: Efficacy of Simplifying Complex Insulin Regimen on Glycometabolic Parameters and Target Organ Damage in Type 2 Diabetes: A Retrospective Cohort Study
Source: J Diabetes Res. 2025 Apr 15;2025:9141564. doi: 10.1155/jdr/9141564 (PMC12014261; doi:10.1155/jdr/9141564)
Supplement: Supporting Information — Additional supporting information can be found online in the Supporting Information section. Supporting Information S1. In-depth description of the calculation of various statistical indicators on target organ damage and adverse effects outcomes. Proportions were used to express the frequency of outcomes, while relative risk (RR) and relative risk reduction (RRR) demonstrated the proportional differences between groups. Absolute risk reduction (ARR) provided the direct difference in event occurrence between the treatment and control groups. The number needed to treat (NNT) quantified how many individuals need to be treated to achieve one favorable outcome. The odds ratio (OR) was used to compare the relative odds of events, particularly for rare outcomes. All estimates were presented with 95% confidence intervals (CIs) to ensure the accuracy and reliability of the evaluations. Table S2. Clinicopathologic characteristics of patients in the simplification group at the BV visit. Patients were grouped based on whether therapy simplification was conducted with iGlarLixi or with IDegLira. Student's t-test and the chi-square test were used as appropriate. Data are presented as medians with 25th and 75th percentiles. p values of < 0.05 were considered to indicate statistical significance. Figure S3. Changes of major clinical parameters of patients in the simplification group from the BV to the 24-month visit. The figure shows HbA1c (a), body weight (b), and fixed-ratio combination (FRC) doses in U/day (c) or in U/kilogram/day (d). Patients were grouped based on whether therapy simplification was conducted with iGlarLixi (empty circle) or with IDegLira (black circle). All data are displayed as medians with 25th and 75th percentiles, indicated as straight lines. Analyses within treatment arms compared to the baseline visit (BV) were performed using repeated measures analysis of variance test followed by Dunn's method. Comparisons between subgroups within specific time points [file 9141564.f1.docx]

**Supplementary Material S1. In-depth description of the calculation of various statistical indicators on target organ damage and adverse effects outcomes.** Proportions were used to express the frequency of outcomes, while relative risk (RR) and relative risk reduction (RRR) demonstrated the proportional differences between groups. Absolute risk reduction (ARR) provided the direct difference in event occurrence between the treatment and control groups. The number needed to treat (NNT) quantified how many individuals need to be treated to achieve one favorable outcome. The odds ratio (OR) was used to compare the relative odds of events, particularly for rare outcomes. All estimates were presented with 95% confidence intervals (CIs) to ensure the accuracy and reliability of the evaluations.

**Relative risk:**

$$RR=\frac{P_{treatment}}{P_{control}}$$

where

$$P_{treatment}=\frac{Number of patients with event during therapy simplification}{Number of patients with therapy simplification}$$

$$P_{control}=\frac{Number of patients with event with ongoing ICT}{Number of patients with ongoing ICT}$$

**Relative risk reduction:**

$$RRR=1-RR$$

**Absolute risk reduction:**

$$ARR=P_{control}-P_{treatment}$$

**Number needed to treat:**

$$NNT=\frac{1}{ARR}$$

**Odds ratio:**

$$OR=\frac{A \times D}{B \times C}$$

where

A: Number of patients with event during therapy simplification.

B: Number of patients without event during therapy simplification.

C: Number of patients with event with ongoing ICT.

D: Number of patients without event with ongoing ICT.

**Supplementary Table S2. Clinicopathologic characteristics of patients in the simplification group at the BV visit.** Patients were grouped based on whether therapy simplification was conducted with iGlarLixi or with IDegLira**.** Student’s *t* test and the chi-square test were used as appropriate. Data are presented as medians with 25^th^ and 75^th^ percentiles. *P* values of <0.05 were considered to indicate statistical significance.

|  | **iGlarLixi (n=62)** | **IDegLira (n=42)** | ***P* value** |
| --- | --- | --- | --- |
| **Age (years)** | 62.50 (51.75; 68.25) | 66.00 (55.75; 71.00) | 0.086 |
| **Diabetes duration (years)** | 10.50 (3.75; 15.00) | 13.00 (5.00; 32.25) | 0.097 |
| **Male (n, %)** | 28 (45.16%) | 20 (46.51%) | 0.613 |
| **HbA1c (%)** | 8.20 (7.76; 9.00) | 7.80 (6.90; 8.80) | 0.064 |
| **HbA1c (mmol/mol)** | 66.11 (61.47; 74.86) | 61.74 (51.90; 72.67) | 0.064 |
| **C-peptide (ng/mL)** | 3.85 (2.83; 4.80) | 3.50 (2.49; 4.61) | 0.214 |
| **Body weight (kg)** | 93.50 (76.00; 105.25) | 94.50 (79.75; 108.25) | 0.393 |
| **Body mass index (kg/m^2^)** | 32.31 (28.69; 37.19) | 30.00 (26.52; 34.96) | 0.112 |
| **Initial TDD (U/d)** | 53.50 (38.00; 80.50) | 48.00 (42.00; 55.25) | 0.458 |
| **Initial TDD (U/d/kg)** | 0.167 (0.108; 0.211) | 0.163 (0.127; 0.195) | 0.974 |
| **Initial ICT with analog insulins (n, %)** | 33 (53.22%) | 20 (47.62%) | 0.498 |
| **Oral antidiabetics prior to simplifying (n, %)** | | | |
| **Metformin** | 57 (91.93%) | 35 (83.33%) | 0.107 |
| **Sulfonylurea** | 12(19.35%) | 8 (19.05%) | 0.923 |
| **DPP4i** | 17 (27.42%) | 19 (45.23%) | 0.075 |
| **GLP1-RA** | 8 (12.90%) | 3 (7.14%) | 0.329 |
| **SGLT2i** | 22 (35.48%) | 9 (21.42%) | 0.124 |
| **Diabetes-related target organ damage prior to simplifying (n, %)** | | | |
| **eGFR (ml/min/1.73m^2^)** | 82.50 (64.00; 90.00) | 85.00 (67.25; 90.00) | 0.464 |
| **CKD (n, %)** | 11 (17.74%) | 7 (16.66%) | 0.845 |
| **UACR >30 mg/g (n, %)** | 13 (20.96%) | 16 (38.09%) | 0.067 |
| **Retinopathy (n, %)** | 15 (24.19%) | 12 (28.57%) | 0.073 |
| **PSN (n, %)** | 16 (25.81%) | 13 (30.95%) | 0.067 |
| **ASCVD (n, %)** | 17 (27.41%) | 10 (23.81%) | 0.631 |

**Supplementary Figure S3. Changes of major clinical parameters of patients in the simplification group from the BV to the 24-month visit.** Figure shows HbA_1c_ **(a)**, body weight **(b)**, fixed-ratio combination (FRC) doses in U/day **(c)** or in U/kg/day **(d)**. Patients were grouped based on whether therapy simplification was conducted with iGlarLixi (empty circle) or with IDegLira (black circle). All data are displayed as medians with 25^th^ and 75^th^ percentiles, indicated as straight lines. Analyses within treatment arms compared to the baseline visit (BV) were performed using repeated measures analysis of variance test followed by Dunn’s method. Comparisons between subgroups within specific time points were conducted using the Mann–Whitney *U* test, and followed by the Dunn’s *post-hoc* test. **P* < 0.05, ***P* < 0.01, ****P* < 0.001 vs. BV within groups. ^x^*P* < 0.05, ^xx^*P* < 0.01, ^xxx^*P* < 0.001 vs. IDegLira within visit.

**
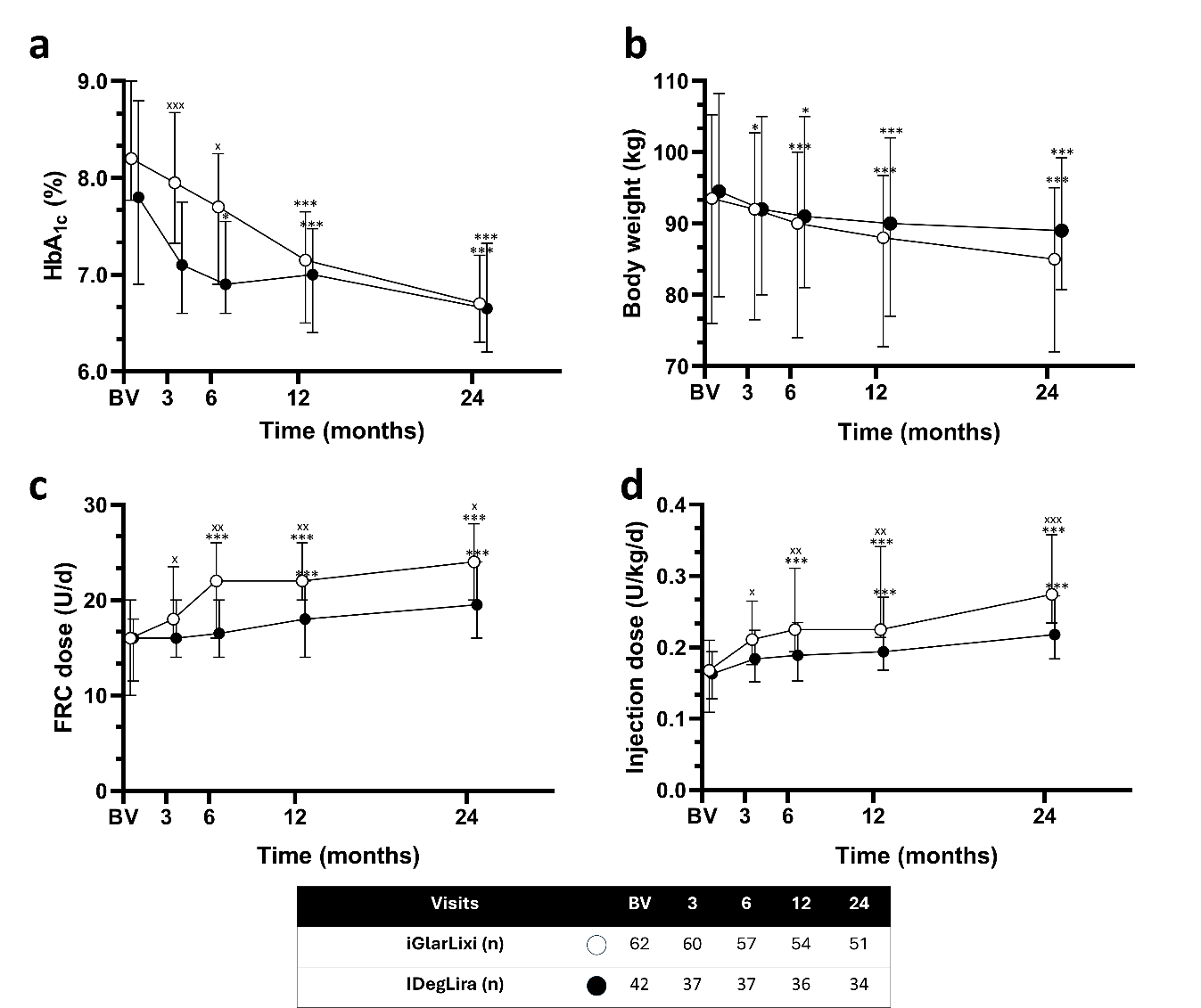
**

**Supplementary Table S4. Risks of target organ damage and hypoglycemia between the control and the simplification group at the 24-month visit.**

|  | **Retinopathy** | **Peripheral sensory neuropathy** | **ASCVD** | **CKD** | **eGFR worsening** | **Elevated UACR** | **Hypoglycemia** |
| --- | --- | --- | --- | --- | --- | --- | --- |
| **Proportion in Control** | 0.3103 | 0.3965 | 0.5741 | 0.2069 | 0.6034 | 0.4828 | 0.3793 |
| **Lower 95% CI** | 0.2062 | 0.2809 | 0.4416 | 0.1225 | 0.4749 | 0.3593 | 0.2656 |
| **Upper 95% CI** | 0.4380 | 0.5251 | 0.6967 | 0.3277 | 0.7191 | 0.6084 | 0.5080 |
| **Proportion in Simplifying** | 0.3077 | 0.3173 | 0.4000 | 0.2019 | 0.2981 | 0.3269 | 0.1058 |
| **Lower 95% CI** | 0.2272 | 0.2357 | 0.3049 | 0.1360 | 0.2186 | 0.2443 | 0.0601 |
| **Upper 95% CI** | 0.4019 | 0.4119 | 0.5033 | 0.2890 | 0.3919 | 0.4218 | 0.1795 |
| **ARR** | 0.0027 | 0.0792 | 0.1741 | 0.005 | 0.3054 | 0.1558 | 0.2735 |
| **Lower 95% CI** | -0.1378 | -0.0702 | 0.0061 | -0.1163 | 0.1463 | 0.0001 | 0.1380 |
| **Upper 95% CI** | 0.1536 | 0.2315 | 0.3292 | 0.1426 | 0.4457 | 0.3062 | 0.4101 |
| **RR** | 0.9915 | 0.8002 | 0.6968 | 0.9760 | 0.4940 | 0.6772 | 0.2788 |
| **Lower 95% CI** | 0.6136 | 0.5234 | 0.4951 | 0.5186 | 0.3442 | 0.4615 | 0.1458 |
| **Upper 95% CI** | 1.6021 | 1.2234 | 0.9807 | 1.8368 | 0.7089 | 0.9936 | 0.5334 |
| **z statistics of RR** | 0.035 | 1.029 | 2.072 | 0.075 | 3.827 | 1.993 | 3.859 |
| **P value of RR** | 0.972 | 0.3034 | **0.0383** | 0.9399 | **0.0001** | **0.0463** | **0.0001** |
| **RRR** | 0.0085 | 0.1198 | 0.3032 | 0.0240 | 0.5060 | 0.3228 | 0.7212 |
| **Lower 95% CI** | -1.6021 | -0.2234 | 0.0193 | -0.8368 | 0.2911 | 0.0064 | 0.4666 |
| **Upper 95% CI** | 0.3864 | 0.4766 | 0.5049 | 0.4814 | 0.6558 | 0.5385 | 0.8542 |
| **NNT** | 377 | 13 | 6 | 201 | 3 | 6 | 4 |
| **Lower 95% CI** | -7 | -14 | 164 | -9 | 2 | 10528 | 7 |
| **Upper 95% CI** | 7 | 4 | 3 | 7 | 7 | 3 | 2 |
| **OR** | 0.9877 | 0.7073 | 0.4946 | 0.9699 | 0.2791 | 0.5204 | 0.1935 |
| **Lower 95% CI** | 0.4930 | 0.3624 | 0.2494 | 0.4378 | 0.1423 | 0.2695 | 0.0853 |
| **Upper 95% CI** | 0.9787 | 1.3805 | 0.9809 | 2.1488 | 0.5471 | 1.0050 | 0.4393 |

**Supplementary Table S5. Changes in medication use at BV and at the 24-month visit.** Comparison was conducted between control and simplification group within visit and between BV and 24-month visit within the treatment group by the chi-square test.

|  | **Control** | | **Simplifying (n=104)** | |
| --- | --- | --- | --- | --- |
|  | **At BV (n=58)** | **At 24-month visit (n=58)** | **At BV (n=104)** | **At 24-month visit (n=90)** |
| **Oral antidiabetics prior to simplifying (n, %)** | | | | |
| **Metformin** | 50 (86.20%) | 50 (86.20%) | 92 (88.46%) | 83 (92.22%) |
| **Sulfonylurea** | 15 (25.86%) | 19 (32.75%) | 20 (19.23%) | 13 (14.44%) |
| **DPP4i** | 23 (39.65%) | 26 (44.83%) | 36 (34.61%) | 0 (0%) |
| **GLP1-RA** | 5 (8.62%) | 5 (8.62%) | 11 (10.57%) | 0 (0%) |
| **SGLT2i** | 23 (39.65%) | 23 (39.65%) | 31 (29.80%) | 24 (26.66%) |
| **Concomitant drug use (n, %)** | | | | |
| **RAASi** | 42 (72.41%) | 46 (79.31%) | 67 (64.42%) | 63 (70.00%) |
| **Beta blockers** | 26 (44.82%) | 33 (56.89%) | 58 (55.77%) | 55 (61.11%) |
| **MRA** | 14 (24.13%) | 17 (29.31%) | 34 (32.69%) | 30 (33.33%) |
| **Statins** | 33 (56.89%) | 38 (65.51%) | 50 (48.07%) | 51 (56.66%) |
| **Antiplatelet agents** | 20 (34.48%) | 29 (50.00%) | 39 (37.50%) | 42 (46.66%) |

**Supplementary Material S6. Comparison of cardiorenal effects of liraglutide and lixisenatide with a brief comparison of LEADER and ELIXA cardiovascular outcome trials.**

The LEADER trial (NCT01179048) was a groundbreaking study that assessed the cardiovascular benefits of liraglutide in patients with T2D (including HbA_1c_ 7.0-10.0%) and documented history of CV disease. According to glycemic control and body weight (BW), administration of liraglutide resulted in an average HbA_1c_Δ of -0.40 (95% CI -0.45 – -0.34; *p* < 0.001) and in an average BWΔ of -2.3 kg (95% CI -2.5 – -2.0; *p <* 0.001). The results demonstrated a significant reduction in the risk of major adverse cardiovascular events (MACE) by 13% compared to placebo (HR 0.87; 95% CI 0.78–0.97; *p* = 0.01). Additionally, either the cardiovascular mortality, or all-cause mortality reduced significantly (HR 0.78; 95% CI 0.66–0.93; *p* = 0.007; HR 0.85; 95% CI 0.74–0.97; *p* = 0.02, respectively). Liraglutide also provided a significant renal protection, showing a 22% reduction in adverse renal outcomes, primarily due to a lower incidence of new-onset persistent macroalbuminuria (HR 0.78; 95% CI 0.67–0.92; *p* = 0.003). While no significant difference was found in hospitalization for heart failure (HR 0.87; 95% CI 0.73–1.05; *p* = 0.14), the overall cardiovascular and renal benefits firmly positioned liraglutide as a valuable therapy.

On the other hand, the ELIXA trial (NCT01147250) was a cardiovascular outcomes study designed to evaluate the safety of lixisenatide in T2D patients who had experienced an acute coronary event within the previous 180 days. The trial demonstrated that lixisenatide was non-inferior to placebo for MACE, with a hazard ratio of 1.02 (95% CI: 0.89–1.17; *p* = 0.81), confirming its cardiovascular safety. Lixisenatide also modestly improved glycemic control, resulting in an average reduction in HbA_1c_ of -0.27% compared to placebo by the end of the trial (*p* < 0.001). Additionally, participants treated with lixisenatide experienced a small but significant reduction in body weight, averaging -0.7 kg (95% CI: -0.9 to -0.5 kg; *p* < 0.001). Importantly, lixisenatide did not change the risk of hospitalization for heart failure, with a HR of 0.96 (95% CI: 0.75–1.23). The findings confirmed the cardiovascular safety of lixisenatide but did not demonstrate significant cardiovascular benefits beyond glycemic control. While lixisenatide showed modest reductions in HbA_1c_ and body weight, it lacked the additional cardiovascular protective effects seen with liraglutide. This positioned lixisenatide primarily as a safe option for glycemic control in patients recovering from an acute coronary event, rather than as a treatment for reducing cardiovascular risk. The lack of significant CV benefit observed in the ELIXA trial, compared to LEADER trial, can be attributed to several factors. Lixisenatide is a short-acting GLP1-RA, mainly controlling postprandial glucose, whereas long-acting agents like liraglutide offer sustained receptor activation, which may lead to broader metabolic and cardiovascular benefits. Additionally, lixisenatide’s shorter duration of action may limit its effects on vascular function and inflammation. The ELIXA trial also focused on patients with advanced atherosclerotic disease following an acute coronary syndrome, a population less likely to benefit from further cardiovascular protection. Moreover, lixisenatide may lack some of the pleiotropic cardiovascular effects seen with other GLP-1 receptor agonists, which could explain the differences in outcomes. Finally, the trial's shorter duration and primary focus on safety rather than efficacy may have limited the detection of long-term cardiovascular benefits. These factors likely contributed to the lack of observed cardiovascular benefits with lixisenatide.

**Supplementary Material S7. Representative comparison of publications examining the effects of IDegLira or iGlarLixi in a simplifying approach.**

| **Authors** | **Examined FRC** | **Length** | **Study design** | **HbA1c change in % (mean)** | **Body weight change in kg (mean)** | **Hypoglycemic episodes (% of patients)** | **Adverse events (% of patients)** | **Final FRC dose in units/day (mean)** |
| --- | --- | --- | --- | --- | --- | --- | --- | --- |
| Taybani et al.[1] | IDegLira | 12 weeks | prospective, observational cohort study | -0.3 | -3.11 | 45 | n/a | 20.76 |
| Zenari et al. [2] | IDegLira | 48 weeks | retrospective observational cohort study | -0.2 | -6.2 | 0 | 1.72 | 25.9 |
| Taybani et al. [3] | IDegLira | 48 weeks | prospective, observational cohort study | -0.2 | -3.8 | 8.6 | 25 | 21.9 |
| Persano et al. [4] | IDegLira | 24 weeks | prospective, observational cohort study | -0.6 | -2.4 | n/a | n/a | 22.0 |
| Martinka et al. [5] | IDegLira | 28 weeks | prospective, observational cohort study | -1.0 | -3.8 | 38.2 | n/a | n/a |
| Romano et Sella [6] | IDegLira | 48 weeks | retrospective patient chart review | -1.2 | -2.0 | 0 | n/a | 24.1 |
| Pantalone et al. [7] | iGlarLixi | 48 weeks | retrospective observational cohort study | -0.6 | n/a | 16.32 | n/a | n/a |
| Ishii et al. [8] | iGlarLixi | 24 weeks | prospective, observational cohort study | + 0.3 | -0.7 | 16.7 | 27.3 | 10.7 |

1. Taybani Z, Bótyik B, Katkó M, Gyimesi A, Várkonyi T. Simplifying Complex Insulin Regimens While Preserving Good Glycemic Control in Type 2 Diabetes. *Diabetes Ther*. 2019;10(5):1869-1878. doi:10.1007/s13300-019-0673-8
2. Zenari L, Da Porto A, De Moliner L, et al. Real-World Evaluation of Glycemic Outcomes and Extra-Glycemic Parameters in Diabetic Patients Treated with the Combined Formulation Degludec-Liraglutide.*Diabetes Ther.* 2021;12(1):197-209. doi:10.1007/s13300-020-00945-4
3. Taybani Z, Bótyik B, Gyimesi A, Katkó M, Várkonyi T. One-year safety and efficacy results of insulin treatment simplification with IDegLira in type 2 diabetes. *Endocrinol Diabetes Metab.* 2023;6(1):e390. doi:10.1002/edm2.390
4. Persano M, Nollino L, Sambataro M, et al. Real-world study on the effectiveness and safety of basal insulin IDegLira in type 2 diabetic patients previously treated with multi-injective insulin therapy. Eur Rev Med Pharmacol Sci. 2021;25(2):923-931. doi:10.26355/eurrev_202101_24661
5. Martinka E, Dravecká I, Tkáč I. Switching from Multiple Insulin Injections to a Fixed Combination of Degludec and Liraglutide in Patients with Type 2 Diabetes Mellitus: Results from the Simplify Study After 6 Months. Diabetes Ther. 2023;14(9):1503-1515. doi:10.1007/s13300-023-01435-z
6. Romano I, Serra R. Use of IDegLira to Intensify, Simplify, and Increase Appropriateness of Type 2 Diabetes Therapy: A Real-Life Experience [published correction appears in Diabetes Ther. 2024 Jun;15(6):1483. doi: 10.1007/s13300-024-01576-9]. Diabetes Ther. 2024;15(1):145-154. doi:10.1007/s13300-023-01489-z
7. Pantalone KM, Heller C, Lajara R, et al. Initiation of iGlarLixi Versus Basal-Bolus Insulin in Adults With Type 2 Diabetes Advancing From Basal Insulin Therapy: The SoliComplex Real-World Study. Diabetes Spectr. 2023;36(3):253-263. doi:10.2337/ds22-0064
8. Ishii H, Kamiya H, Takahashi Y, Morimoto Y, Yabe D. Quality of Life in Japanese People with Type 2 Diabetes Switching from Multiple Daily Insulin Injections to Once-Daily iGlarLixi: SIMPLIFY Japan. Diabetes Ther. 2024;15(11):2381-2400. doi:10.1007/s13300-024-01645-z
